# Study on Effect of Shapes of Serration of Joining Plane on Joining Characteristics for the Aluminum–Steel Multi-Materials Press Joining Process

**DOI:** 10.3390/ma13245611

**Published:** 2020-12-09

**Authors:** In-Kyu Lee, Sung-Yun Lee, Sang-Kon Lee, Myeong-Sik Jeong, Bong-Joon Kim, Won-Gwang Joo

**Affiliations:** 1 Mechanical Components and Materials R&D Group, Korea Institute of Industrial Technology, 320 Techno sunhwan-ro, Yuga-eup, Dalseong-gun, Daegu 42994, Korea; lik1025@kitech.re.kr (I.-K.L.); yunskills@kitech.re.kr (S.-Y.L.); msjeong@kitech.re.kr (M.-S.J.); 2 Central R&D Institute, Kyung Chang Industrial Co., Ltd., Seongseo-ro 35-gil 6, Dalseo-gu, Daegu 42719, Korea; bulkkot@empas.com (B.-J.K.); wgjoo@kc.co.kr (W.-G.J.)

**Keywords:** press joining process, aluminum–steel multi-material, interfacial serration, joining force, torque

## Abstract

Recently, mechanical joining processes have received much attention for joining multi-materials. In particular, these processes have a great demand in the automobile industry for weight reduction. The press-fitting process is a representative mechanical joining process. In this process, the shape of the interfacial serration on the joining plane is very important because it has a significant effect on the joining strength. In this study, the characteristics of the aluminum–steel press joining process were investigated according to the shape of the interfacial serration of the joining plane. The deformation of the material and the forming load were investigated by conducting finite element analysis. In addition, the unfilled height of the material, joining force, and torque were measured experimentally.

## 1. Introduction

Transporting machines, such as automobiles and aircraft, are made up of numerous parts. Automobiles are assembled using more than 10,000 to 30,000 parts [1]. Recently, many studies have been carried out to reduce the weight of automobiles to improve fuel efficiency [2]. The development of ultra-high-strength steel has been actively carried out, and many studies have been recently conducted on the application of lightweight materials such as aluminum, magnesium, and carbon fiber composites [3,4,5,6,7]. In addition, various studies are being carried out on the joining processes to combine steel and lightweight materials in accordance with the expansion of the application of lightweight materials. Lightweight automotive construction depends not only on the lightweight materials but also on suitable joining methods for multi-material combinations with high process reliability [8]. It is difficult to join multi-materials using conventional welding processes because of the difference between thermal and mechanical properties. To overcome this problem, many studies on mechanical joining processes have been actively carried out. Raoelison et al. investigated the elaboration of a weldability window of an aluminum alloy 6060T6 tubular assembly welded using magnetic pulse welding [9]. Chastel et al. described several mechanical joining processes, including self-piercing rivet, clinching, and high-speed joining [10]. Lee et al. investigated the effects of process parameters of the mechanical clinching process on the joint characteristics of advanced high-strength steel DP780 and Al5052 alloy sheets [11]. Busse et al. proposed a sheet pre-punched clinching process to increase the range of materials for clinching [12]. Maschut et al. investigated the characteristics of friction element welding, resistance spot welding, resistance element welding, and self-piercing rivet [8]. Higgins explained the adhesive bonding used in commercial aircraft [13].

In recent times, studies have been actively carried out on the joining of multi-materials using a cold-forming process. Wagener and Haats studied the cold pressure welding of aluminum and titanium tube using an extrusion process [14]. Groche and Tibari studied a hydroforming process to join hollow tubes without a welding process [15]. Alves et al. introduced the flexible joining of sheet panels to thin-walled tubular profiles by means of a two-stage tube-end forming process [16]. Jäger et al. developed a combined joining process of deep drawing and cold forging to produce sheet and bulk metal parts [17].

**Figure 1:**
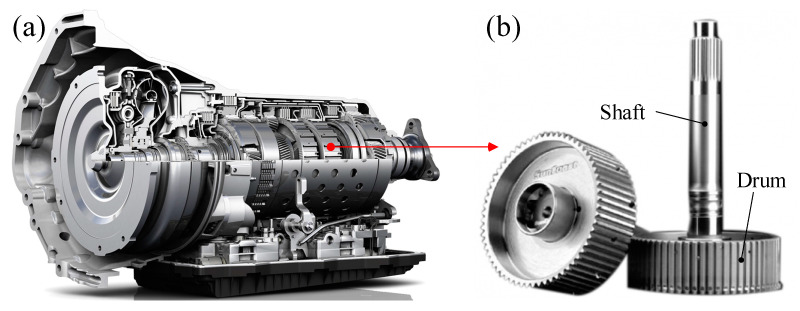
Automotive transmission and clutch drum. (**a**) Auto transmission; (**b**) clutch drum.

As shown in Figure 1, the automotive transmission is very complex and consists of various components. The clutch drum is a very important component that transmits a turning moment from the clutch to a driving device. As shown in Figure 1b, the clutch drum consists of a shaft and a drum. The drum has a tooth on the circumference for power transmission.

In general, after the shaft and drum are manufactured from carbon steel, the two parts are joined with a joining process. The clutch drum requires high torsional strength and high rotational accuracy. In addition, lightweight is very important to improve fuel efficiency [18]. Therefore, it is very effective to apply lightweight materials to the drum because it can reduce the large moment of inertia. When steel shafts and aluminum drums are applied, joining is very important. Recently, several studies have been carried out on the cold joining process using plastic deformation. Kitamura et al. developed a plastic cold joining method for a rotor shaft with a flange for automobile axle parts [19]. In this study, a high-strength shaft with serrated teeth was indented into the hole of a thick disc at room temperature. Hirota et al. assembled a stepped shaft and disc by indenting the disc with a stepped and grooved shaft up to a certain depth [20] and described the effect of the penetration depth of the stepped portion of the shaft on the cavity filling behavior for the mechanical joining of the shaft and holed disc [21]. Afonso et al. presented a new joining by the boss forming process for fixing rods and tubes to sheets [22]. Kleditzsch et al. investigated the joining process of steel-aluminum knurled interference fits [23]. They provided detailed investigation results of the influence of the shaft-chamber angle, hub-diameter ratio, and geometric interference on knurled interference fits.

In this study, the authors investigated the effect of five different shapes of serration on the press joining load, axial joining strength, and torque in the press joining process of a steel shaft and an aluminum drum. The deformed shape and forming load were investigated by conducting finite element (FE) analysis. Then, the deformed shape of the material, unfilled ratio, axial joining strength, and torque were evaluated experimentally.

## 2. Materials and Methods

### 2.1. Materials

**Figure 2:**
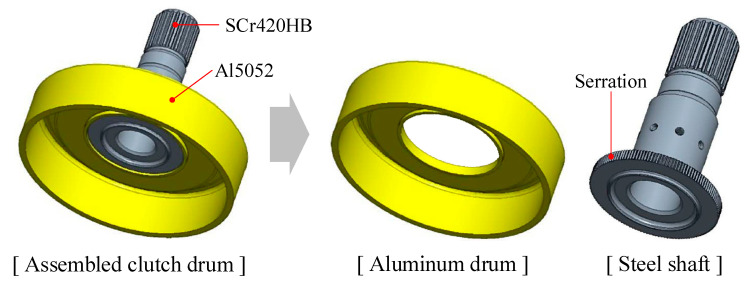
Multi-material clutch drum.

As shown in Figure 2, the multi-material clutch drum consists of a steel shaft and an aluminum drum. In this study, the shaft and drum materials were SCr420 and Al5052, respectively. FE analysis was carried out to investigate the material flow and forming load of the press joining process. The shaft and drum are assumed to be rigid and elastoplastic materials, respectively [18]. The material properties of Al5052 for the drum were obtained by conducting a tensile test. Table 1 shows the mechanical properties of Al5052.

### 2.2. Effect of Shapes of Serration on Joining Plane

**Figure 3:**
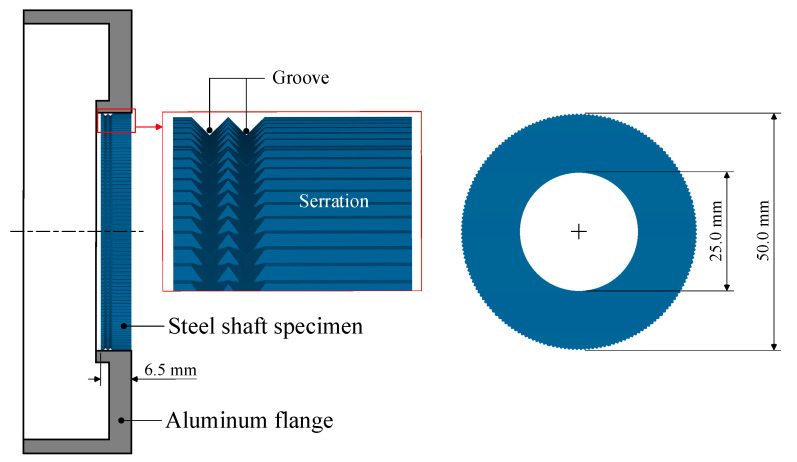
SCr420 inner part for joining.

As shown in Figure 3, a doughnut-shaped specimen was used, considering the joining plane of the steel shaft. To increase the joining strength, not only the serration but also two grooves were machined on the joining plane.

**Figure 4:**
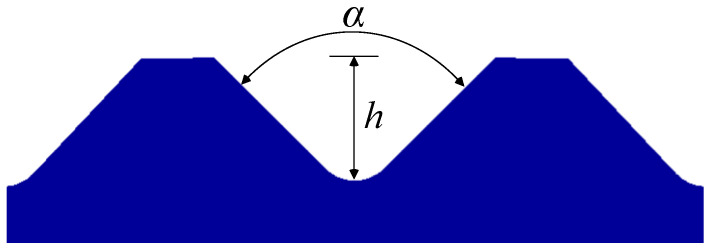
Angle (α) and height (*h*) of the serration of steel inner.

In this study, five different shapes of serration were used to investigate the effect of the shape on the joining characteristics. As shown in Figure 4, the angle and the height of the serration vary with five different shapes. Table 2 shows the applied shapes of the serration, with number 5 being diamond-shaped.

### 2.3. Elastoplastic FE Analysis of Joining Process

**Figure 5:**
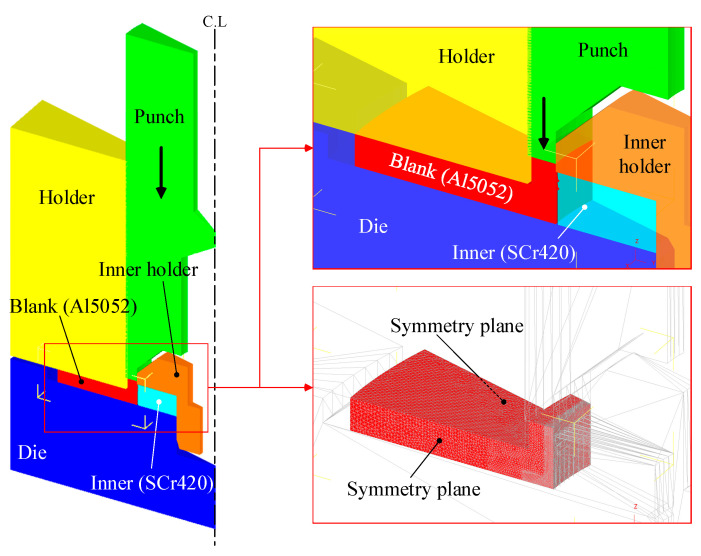
Initial finite element (FE) analysis model.

To analyze the material flow and forming load of the press joining process, FE analysis was carried out using DEFORM 3D (Ver. 12). As shown in Figure 5, a 1/24 model was used, considering the axisymmetric condition. The deformable aluminum blank is an elastoplastic material, and the other tools are assumed to be rigid bodies. The numbers of the element meshes and nodes are 198,138 and 43,409, respectively.

**Figure 6:**
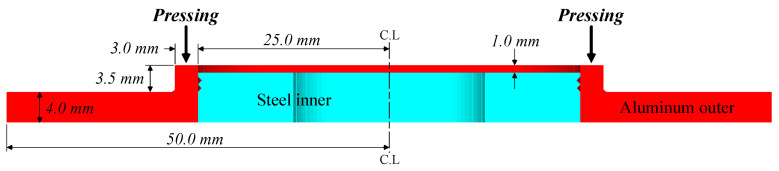
Shape and dimensions of aluminum outer.

Figure 6 shows the shape and dimensions of the aluminum outer. As shown in Figure 6, the upper part of the boss of the aluminum outer is pressed to join the steel inner and the aluminum outer.

The friction coefficient (μ) between the blank and the tools was assumed to be 0.35, considering non-lubrication [24]. The FE analysis conditions are listed in Table 3.

### 2.4. Evaluation of Joining Force

**Figure 7:**
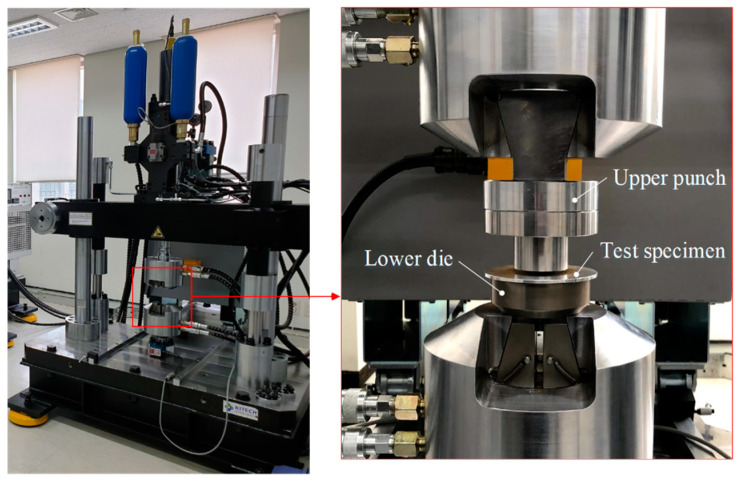
Experiment for measuring the joining force.

In this study, the axial joining force was measured to evaluate the effect of the shapes of serration on the joining quality using a 20-ton capacity material test machine. Figure 7 shows the experimental equipment.

## 3. Results and Discussion

### 3.1. Knurling Process

In this study, the serration on the joining plane of the steel inner was fabricated using a knurling process. Table 4 shows the fabricated serrations and their dimensions. As shown in Table 4, the measured heights of the five serrations are smaller than the target heights. The differences are 0.06, 0.05, 0.06, 0.05, and 0.06 mm, respectively.

### 3.2. Result of FE Analysis

**Figure 8:**
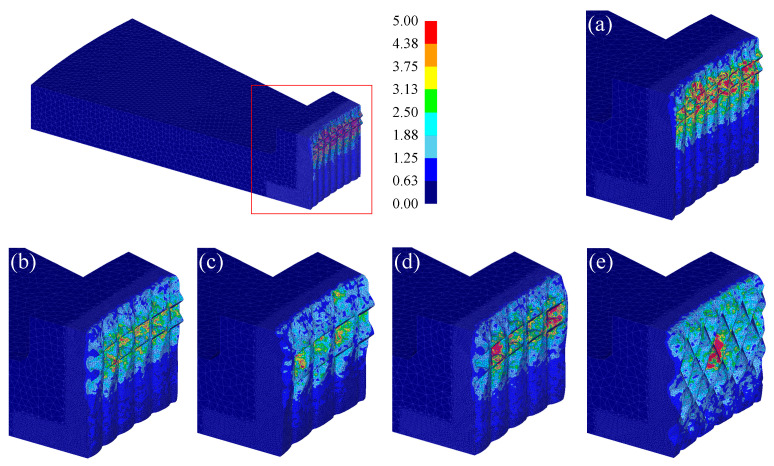
Distribution of effective strain: (**a**) Case 1; (**b**) Case 2; (**c**) Case 3; (**d**) Case 4; (**e**) Case 5.

Figure 8 shows the distribution of effective strain after the forming process (punch stroke: 1.0 mm). The maximum strain occurs at the intersection of the serration and groove, where the deformation is most severe.

**Figure 9:**
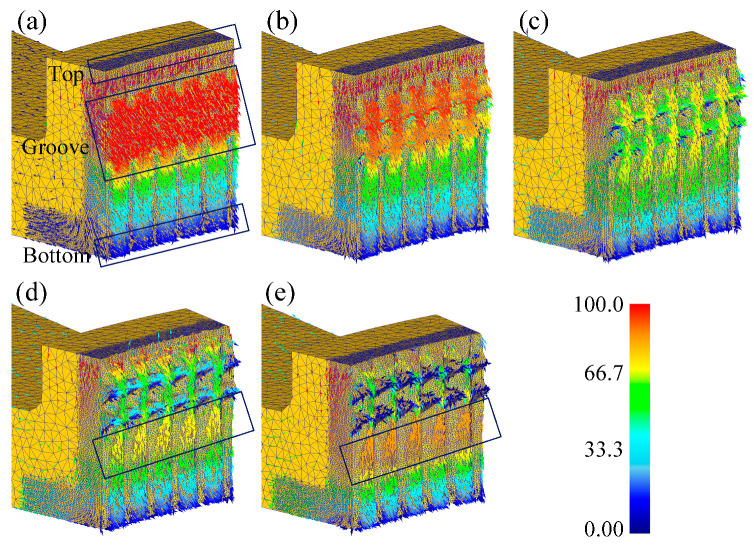
Distribution of velocity (mm/s) (Case 2): (**a**) stroke 0.100 mm; (**b**) stroke 0.260 mm; (**c**) stroke 0.495 mm; (**d**) stroke 0.755 mm; (**e**) stroke 1.000 mm.

Figure 9 shows the distribution of velocity according to the punch stroke. At the early forming stage (Figure 9a,b), the velocity near the grooves is faster than in other regions because of the large cavity in the grooves. As shown in Figure 9c, the velocity deviation decreases after the grooves are filled because of the flow restriction in the grooves. Subsequently, the velocity under the groove increases to fill the serrations, as shown in Figure 9d,e. It can be observed that the velocity near the bottom is always lower than in other regions because the bottom is farther from the punch.

**Figure 10:**
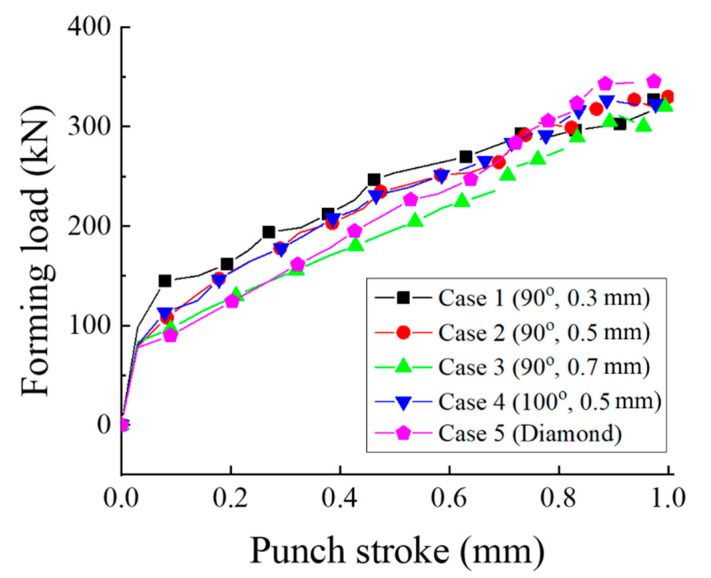
Forming load with punch stroke (FE analysis).

Figure 10 shows the variation in the forming load with the punch stroke. The forming load increases gradually with the punch stroke. At the final forming stage, the forming loads are 331.1, 329.8, 323.6, 330.49, and 359.3 kN. Case 5 has the highest forming load because the cavity of the groove and the serration are the least. If the serration angle is the same, the forming load increases with the decrease in serration height because the decrease in serration height increases the restriction on material flow after cavity filling. In addition, from the comparison between Cases 2 and 4, if the serration height is the same, the serration angle has little effect on the forming load.

### 3.3. Joining Experiment

**Figure 11:**
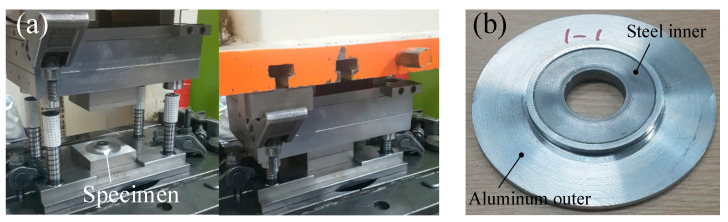
Die set and fabricated specimen: (**a**) die set; (**b**) specimen.

A joining process die set was manufactured to fabricate the joined specimen. Figure 11 shows the forming die set and the fabricated specimen.

**Figure 12:**
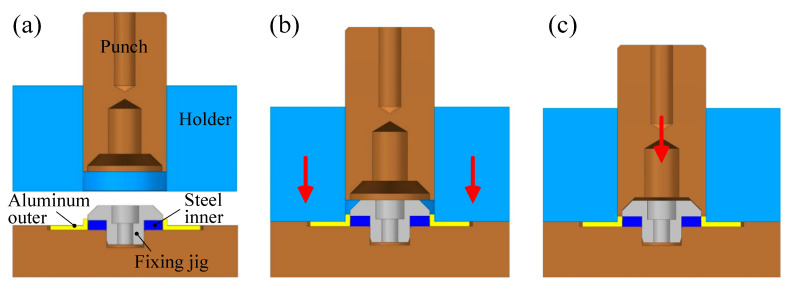
Die set operation: (**a**) initial setting; (**b**) outer holding; (**c**) press forming.

Figure 12 illustrates the operation of the die set. There is a fixing jig at the center to maintain concentricity. As shown in Figure 12b, the outer is held by the holder. Subsequently, the outer and the inner are combined through the movement of the punch.

**Figure 13:**
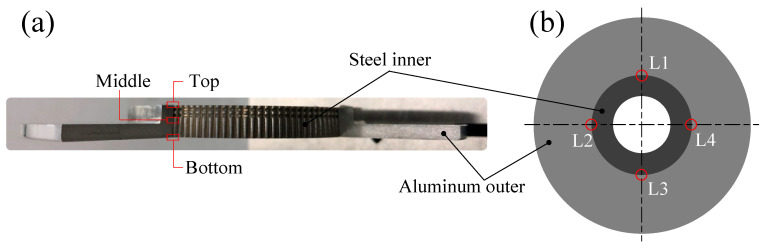
Fabricated specimen: (**a**) joined specimen; (**b**) measured points.

The unfilled height was measured after the joining experiment. The average value was calculated after measuring the unfilled height of the four locations at intervals of 90°, as shown in Figure 13b.

**Figure 14:**
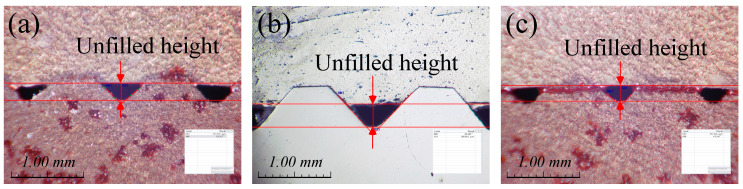
Cross section of L3 of Case 2: (**a**) top; (**b**) middle; (**c**) bottom.

Figure 14 shows the photo of L3 of Case 2 as a representative. It can be observed that the unfilled height of the middle is lower than those of the top and bottom. The measured average value of the unfilled height is summarized in Table 5. It can be observed that the unfilled height of the middle is relatively low in all cases because the material filling occurs first in the grooves, as mentioned in Figure 9. In addition, the unfilled height of the top is lower than that of the bottom because the material flow of the top near the forming punch is faster than that of the bottom. Therefore, if a double-action press that can compress the top and bottom simultaneously is applied, it is thought that the unfilled height at both the top and bottom can be reduced.

### 3.4. Evaluation of Joining Force

**Figure 15:**
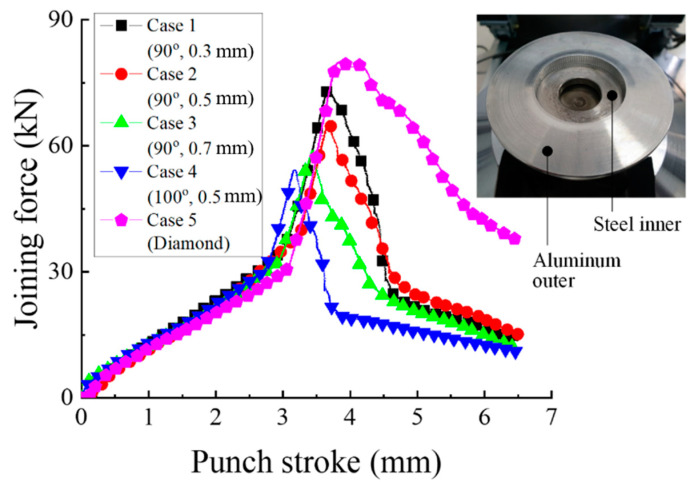
Joining force vs. punch stroke.

Figure 15 shows the measured joining force and specimen. The experiment was carried out until the inner and outer parts were completely separated. The maximum joining forces are 73.4, 65.4, 58.9, 54.3, and 80.0 kN. From Case 1 to Case 4, the force increases gradually and then rapidly decreases after the maximum force. However, in Case 5, the force gradually decreases after the maximum force because there are intersected diamond-shaped serrations on all joining interfaces.

**Figure 16:**
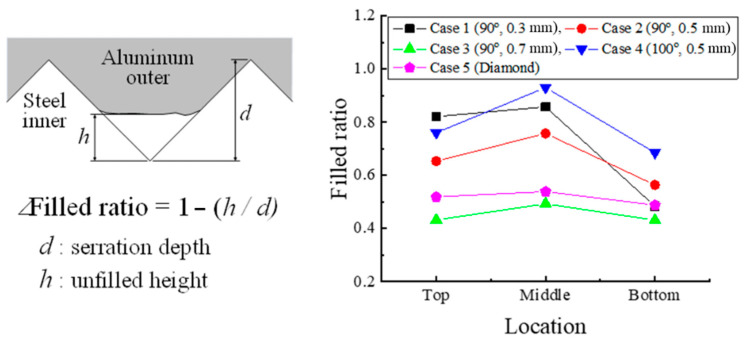
Filled ratio.

Figure 16 shows the filled ratio. In Cases 1, 2, and 3, the joining force increases as the serration height decreases because of better material filling into the serration. The better material filling increases the friction force and the locking force between the aluminum outer and the steel inner. Therefore, the joining force increases as the filled ratio increases. Case 5, with diamond-shaped serration, has the highest joining force of 80.0 kN. The serrations intersect with each other, and the intersected serrations are fabricated on the joining interface, thereby improving the joining force in Case 5. In addition, the force in Case 5 decreases relatively less than that in the other cases because of the intersected diamond-shaped serration on the joining interface.

### 3.5. Evaluation of Joining Torque

The joining torque of the specimens was measured using a dial-torque wrench (Model: EDS 1400S). Table 6 shows the measured maximum torque. We can expect that the torque to increase with a higher serration height. However, Case 2 has the highest torque for the three cases with the same angle because its filled ratio is higher than that of Case 3, as shown in Figure 16. From the comparison of Cases 2 and 4 with the same serration height, although the filled ratio of Case 4 is higher than that of Case 2, the torque of Case 2 is higher than that of Case 4. This means that the angle is also an important factor. Therefore, in order to investigate the effect of the serration angle, further research is needed in the future.

## 4. Results and Discussion

The multi-material joining of a steel shaft with an aluminum drum using press joining was investigated. The effect of five different shapes of the serration on the material flow, joining force, filled ratio, and torque was investigated by conducting FE analysis and experiments, and the following results were obtained.

- From the results of FE analysis, at the early forming stage, the material velocity near the punch is higher than that near the fixed die. In addition, the material fills the cavity in grooves first because of the low material flow restriction caused by the wide cavity in the groove. Then the material fills serrations. The bottom of the serrations close to the die is filled last because the velocity near the die is always lower than in other regions. When the serration angle is the same, the forming load of the press joining process increases with the decrease in serration height because of the increased material flow restriction. Case 5 with diamond-shaped serration has the largest joining force of 80.0 kN because of the intersected serration.
- When the serration angle is the same, the joining force increases as the serration height decreases because of the better filling of material into the serration. Therefore, it is very important to improve the filled ratio to increase the joining force.
- In addition, the joining torque depends on the filled ratio. To increase the joining torque, the filled ratio should be maximized. In this study, Case 2 is the best condition because the joining torque is maximum due to the relatively high filled ratio. Under the same geometric condition (serration height, angle), we can expect that the higher the filled ratio, the higher the joining load or torque. However, under various geometric conditions, we must consider these conditions simultaneously, and further research must be carried out to do so.
- In this study, the unfilling occurs in all cases. From the result of FE analysis and experiment, the joining characteristics depend mainly on the filled ratio. If the filled ratio is maximized, we can expect excellent joining characteristics. Therefore, further research is needed to maximize the filled ratio through the process optimization of the press joining process.

## Figures and Tables

**Table 1:** Mechanical properties of Al5052.

Item	Value
Young’s modulus (GPa)	70.3
Poisson’s ratio	0.33
Yield strength (MPa)	120.5
Tensile strength (MPa)	206.0
Flow stress (MPa)	$\bar{\sigma }=371.2{\bar{\varepsilon }}^{0.247}$

**Table 2:** Five shapes of serration on the joining plane.

Case	1	2	3	4	5
Shape	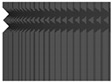	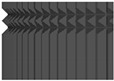	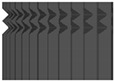	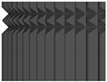	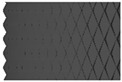
Angle (°)	90	90	90	100	100
Height (mm)	0.3	0.5	0.7	0.5	0.5

**Table 3:** FE analysis conditions.

Conditions	Value
Punch velocity (mm/s)	5.0
No. of elements of blank	144,114
No. of nodes of blank	31,828
Friction coefficient (μ)	0.35

**Table 4:** Dimensions of fabricated serrations.

Case	Shape	Cross Section	Dimension
**1**	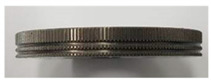	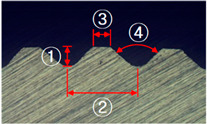	① 0.24 mm ② 0.82 mm ③ 0.19 mm ④ 90°
**2**	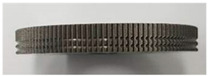	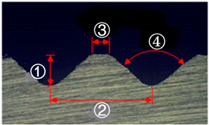	① 0.45 mm ② 1.23 mm ③ 0.19 mm ④ 90°
**3**	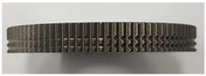	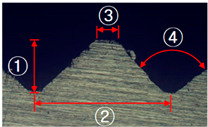	① 0.64 mm ② 1.62 mm ③ 0.21 mm ④ 90°
**4**	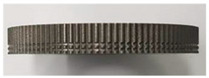	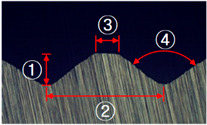	① 0.45 mm ② 1.43 mm ③ 0.27 mm ④ 100°
**5**	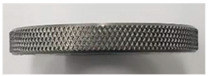	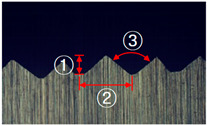	① 0.24 mm ② 0.613 mm ③ 100°

**Table 5:** Measured average unfilled height (mm).

Location	Case 1	Case 2	Case 3	Case 4	Case 5
Top	0.043	0.156	0.344	0.108	0.116
Middle	0.034	0.109	0.325	0.032	0.111
Bottom	0.137	0.197	0.364	0.142	0.123

**Table 6:** Maximum torque (Kn-mm).

Case	Case 1	Case 2	Case 3	Case 4	Case 5
Torque	1025.0	1192.0	1033.0	1142.0	1008.0
